# Detection of *Aedes* (*Fredwardsius*) *vittatus* Mosquitoes, Yucatán Peninsula, Mexico, 2025

**DOI:** 10.3201/eid3111.251358

**Published:** 2025-11

**Authors:** Rahuel J. Chan-Chable, César R. Rodríguez-Luna, Román Espinal-Palomino, Carlos N. Ibarra-Cerdeña

**Affiliations:** Center for Research and Advanced Studies (Cinvestav), Mérida Unit, Mérida, Mexico

**Keywords:** Vector-borne infections, dengue, chikungunya, Zika, Yellow fever, Yucatán Peninsula, emerging arboviruses, invasive mosquitoes, vector competence, Caribbean bridgehead, biologic invasion, Mexico, North America

## Abstract

We report detection of *Aedes* (*Fredwardsius*) *vittatus* mosquitoes in continental North America, in Yucatán, Mexico. Phylogenetic analysis clustered the sequence from mosquitoes collected in Mexico with Caribbean mosquito lineages, suggesting species introduction via the Caribbean. Given its arbovirus competence, urgent inclusion of the *Ae. vittatus* mosquito in surveillance programs is warranted.

Mosquitoborne arboviruses, such as dengue, Zika, chikungunya, and yellow fever, have expanded dramatically over the past 5 decades, driven by urbanization, globalization, and human mobility (*1*). Dengue and chikungunya alone now cause >50 million infections annually, reflecting a 30-fold increase linked to demographic and ecologic change (*2*). Although *Aedes aegypti* and *Ae. albopictus* mosquitoes remain the primary invasive vectors under surveillance and control, other species of epidemiologic relevance are gaining increased attention as potential emerging threats (*3*).

*Ae.* (*Fredwardsius*) *vittatus* is one such mosquito, notable for its expanding range and proven arboviral vector competence (*4*). Described from Corsica, France, in 1977 (*5*), *Ae. vittatus* is now distributed across Africa, the Mediterranean Basin, the Middle East, and South and Southeast Asia, and sporadic detections have been reported in southern Europe and the Caribbean. *Ae. vittatus* mosquitoes are highly adaptable, breeding in both natural and artificial containers, and thrive in sylvatic, rural, agricultural, and periurban environments (*6*). Laboratory and field studies confirm the species’ ability to transmit dengue, chikungunya, Zika, and yellow fever viruses and its additional potential to transmit Japanese encephalitis and West Nile viruses (*7*). 

During entomological surveillance in August–September 2025, we collected 67 adult *Ae.* *vittatus* mosquitoes in traditional Mayan cornfields (milpa) (Appendix Figure 1) on the outskirts of the Mama and Teabo municipalities of Yucatán, Mexico (Table; Figure). We aspirated adult mosquitoes as they attempted to bite field personnel (Appendix Figure 2, panel A). We collected both sexes (Table; Appendix Figure 2, panels B, C), supporting evidence of local reproduction and establishment in rural agricultural environments. 

**Table:** *Aedes* (*Fredwardsius*) *vittatus* mosquitoes collected in the Yucatan Peninsula, Mexico, 2025

Collection date	Collection time	Location	Municipality	State	Latitude, °N	Longitude, °W	Elevation, m*	No./sex
Aug 24	16:00	Mama	Mama	Yucatán	20.480198	−89.357942	23	2/F
	16:30	Mama	Mama	Yucatán	20.480300	−89.357940	22	1/M
Aug 25	06:00–07:00	Mama	Mama	Yucatán	20.480049	−89.357824	23	4/F
Sep 6	16:00–18:00	Mama	Mama	Yucatán	20.480198	−89.357942	23	3/F
	16:00–18:00	Mama	Mama	Yucatán	20.480198	−89.357942	23	5/M
Sep 17	16:00–18:00	Mama	Mama	Yucatán	20.480049	−89.357824	23	33/M
	16:00–18:00	Mama	Mama	Yucatán	20.480049	−89.357824	23	18/F
	16:00–16:30	Teabo	Teabo	Yucatán	20.40315	−89.287062	22	1/M

*Meters above sea level.

**Figure:**
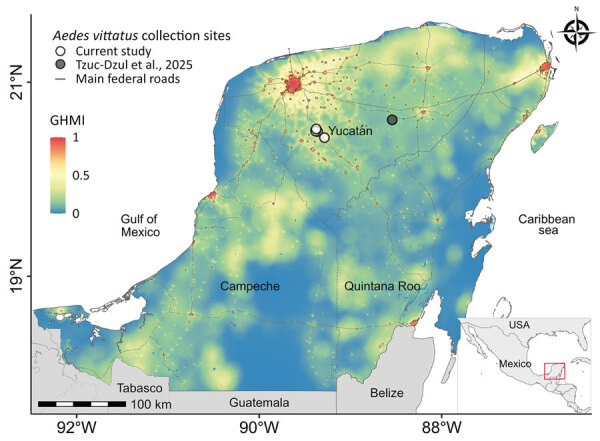
*Aedes* (*Fredwardsius*) *vittatus* mosquito detection and GHMI, Yucatán Peninsula, Mexico, 2025. White circles show sites where mosquitoes were detected in this study; gray circle shows site of mosquito detection from another study (J.C. Tzuc-Dzul et al., unpub. data, https://doi.org/10.21203/rs.3.rs-6786909/v1). Inset shows Mexico with study region marked. Map is overlaid with the GHMI (0.09 km^2^ resolution). GHMI measures landscape modified by humans and values range from 0 (unmodified) to 1 (completely modified). Gray lines indicate main federal and state roads. GHMI, Global Human Modification Index.

We morphologically identified specimens by using standard taxonomic keys (*4*,*5*) and deposited 7 voucher individuals (nos. AR-0734–40), in the Arthropod Collection (ECOSUR, https://colecciones.ecosur.mx), Chetumal Unit. *Ae. vittatus* mosquitoes can be distinguished from other *Aedes* species mosquitoes by their dark proboscis with pale yellowish scales, small bilateral patches of white scales on the clypeus, 3 pairs of narrow white patches on the anterior scutum, a short maxillary palp with apical white scaling, and a distinct white patch at the midpoint of the third tibia (Appendix Figure 2, panels B–E). 

To confirm species identity, we sequenced a fragment of the mitochondrial cytochrome oxidase 1 (COX1) gene from an *Ae.* *vittatus* mosquito we collected in Yucatán, Mexico (GeneBank accession no. PX418072), and analyzed it with global reference sequences. Bayesian phylogenetic inference placed the mosquito specimen from Mexico within the American–Caribbean lineage, clustering with sequences from Cuba and the Dominican Republic (Appendix Figure 3). Although the history of *Ae. vittatus* mosquito invasion is only beginning to unfold, this regional pattern resembles the early stages of *Ae. aegypti* mosquito expansion, for which the Caribbean acted as a bridgehead before dispersal into the Americas and beyond (*8*). Although the 0.8–0.9 posterior support for the *Ae. vittatus* subclade in North America was moderate, the overall tree was well resolved (Appendix Figure 3), strengthening confidence in this inference. The case of *Ae. aegypti* mosquitoes illustrates how the Caribbean can serve as an intermediate launch point for Old World mosquitoes, underscoring the importance of acting now to monitor *Ae. vittatus* mosquitoes and prevent wider establishment as a new invasive vector in the Americas.

We also characterized the ecologic context of the *Ae. vittatus* mosquito using the Global Human Modification Index (The Nature Conservancy, https://gdra-tnc.org/current). High human modification index scores in the Yucatán Peninsula reflect intense land-use change from urbanization, agriculture, and infrastructure projects, highlighting conditions favorable for mosquito establishment and spread (Figure). As a flat landmass with few natural biogeographic barriers, the peninsula provides little resistance to dispersal of habitat-tolerant invasive species. Studies of *Ae. aegypti* mosquitoes have shown that flat, highly connected regions with dense human activity enhance mosquito gene flow and facilitate spread (*9*). By analogy, regions where *Ae.* *vittatus* mosquitoes are now reported, including the Yucatán Peninsula, present similar ecologic and sociological conditions that could accelerate its population increase and dispersal.

Detection of *Ae.* *vittatus* mosquitoes in southeastern Mexico highlights the potential emergence of a new arbovirus vector in the Americas. The Yucatán Peninsula is undergoing profound anthropogenic change, where deforestation, agricultural expansion, and large-scale infrastructure projects like the Tren Maya (*10*) are rapidly reshaping landscapes. Beyond their economic and social goals, such megaprojects can intensify ecosystem degradation, reduce ecologic barriers, and enhance human connectivity, thereby creating ideal conditions for the establishment and spread of invasive mosquitoes. Those dynamics underscore the need to integrate health considerations into land-use planning, recognizing that environmental transformation can amplify the risk for vectorborne diseases. 

In conclusion, detection of *Ae.* *vittatus* mosquitoes in continental North America, specifically in Mexico’s Yucatán Peninsula, highlights the species’ ecologic plasticity and the urgent need to investigate introduction pathways and its potential role in arboviral transmission. Including the *Ae. vittatus* mosquito in regional surveillance and control programs will be essential to anticipate its spread and mitigate future public health impacts.

This article was preprinted at https://www.biorxiv.org/content/10.1101/2025.10.29.684036v1.

Appendix Additional information on detection of *Aedes* (*Fredwardsius*) *vittatus* mosquitoes, Yucatan Peninsula, Mexico, 2025.
