# Supplementary material for: Detection of Aedes (Fredwardsius) vittatus Mosquitoes, Yucatán Peninsula, Mexico, 2025
Source: Emerg Infect Dis. 2025 Nov;31(11):2177–9. doi: 10.3201/eid3111.251358 (PMC12704530; doi:10.3201/eid3111.251358)
Supplement: Appendix — Additional information on detection of Aedes (Fredwardsius) vittatus mosquitoes, Yucatan Peninsula, Mexico, 2025. [file 25-1358-Techapp-s1.pdf]

Article DOI: https://doi.org/10.3201/eid3111.251358

EID cannot ensure accessibility for supplementary materials supplied by authors. Readers who have difficulty accessing supplementary content should contact the authors for assistance.

Detection of *Aedes* (*Fredwardsius*) *vittatus* Mosquitoes, Yucatan Peninsula, Mexico, 2025

Appendix

Methods for Molecular Identification of *Aedes* (Fredwardsius) *vittatus*

Nucleic Acid Extraction

Genomic DNA was extracted using the Quick-DNA Miniprep (Zymo Research, USA), following the manufacturer's instructions. Extractions were performed from mosquito legs. The final DNA elution was carried out in 50 µL of DNase/RNase-free water.

Amplification of Cytochrome Oxidase 1 (COX1)

PCR amplifications were performed in a final reaction volume of 25 µL, consisting of 12.5 µL of Phusion High-Fidelity DNA Polymerase (Thermo Fisher Scientific, USA), 1 µL of each primer (10 µM), 7.5 µL of ultrapure water, and 3 µL of genomic DNA. All reactions were conducted in a Veriti 96-well thermal cycler (Applied Biosystems, Foster City, CA, USA).

A fragment of the COX1 gene was amplified using primers LCO-1490 (5′- GGT CAA CAA ATC ATA AAG ATA TTG G - 3′) and HCO-2198 (5′- TAA ACT TCA GGG TGA CCA AAA AAT CA - 3′) (*1*). PCR consisted of an initial denaturation at 94 °C for 1 min; followed by 39 cycles of denaturation at 94 °C for 15 s, annealing at 48 °C for 30 s, and extension at 72 °C for 45 s; with a final extension at 72 °C for 7 min.

Amplicons from PCR assays were resolved by electrophoresis on 1% agarose gels prepared in 1× TAE buffer, stained with RedGel (Biotium, USA), and visualized under UV light using a BioDoc-It2 imaging system (Analytik Jena U.S. LLC, USA). PCR products were sequenced by Sanger sequencing.

Bioinformatic Analyses

Forward and reverse reads were assembled and edited to generate consensus sequences using Geneious Prime v 2025.2.2 (*2*). Sequence alignments were performed using the MAFFT online server v7 (https://mafft.cbrc.jp/alignment/server/) under default parameters (*3*). The resulting alignments were manually reviewed and edited in AliView v1.28 (*4*). The best-fit nucleotide substitution model was selected using jModelTest2, implemented through the CIPRES Science Gateway (https://www.phylo.org/) with default settings (*5*).

Mitochondrial COX1 marker sequences of Aedes spp. were retrieved from GenBank (accession numbers shown in Figure 2) and include representatives from Africa, Asia, Europe, the Caribbean, and the newly generated Mexico sequence.

Phylogenetic relationships among *Aedes vittatus* sequences were inferred using Bayesian Inference (BI) in Geneious Prime v 2025.2.2, applying the GTR + G substitution model and designating *Sorophora vorax* as the outgroup (*6*). Markov Chain Monte Carlo (MCMC) analyses were run for two million generations, with trees sampled every 100 generations. Posterior probabilities were estimated from the sampled trees after discarding the first 25% as burn-in to ensure convergence and stationarity. The final phylogenetic tree was visualized and edited using FigTree v1.4.4 (*7*).

References

1. Folmer O, Black M, Hoeh W, Lutz R, Vrijenhoek R. DNA primers for amplification of mitochondrial cytochrome c oxidase subunit I from diverse metazoan invertebrates. Mol Mar Biol Biotechnol. 1994;3:294–9. PubMed

2. Kearse M, Moir R, Wilson A, Stones-Havas S, Cheung M, Sturrock S, et al. Geneious Basic: an integrated and extendable desktop software platform for the organization and analysis of sequence data. Bioinformatics. 2012;28:1647–9. PubMed https://doi.org/10.1093/bioinformatics/bts199

3. Katoh K, Rozewicki J, Yamada KD. MAFFT online service: multiple sequence alignment, interactive sequence choice and visualization. Brief Bioinform. 2019;20:1160–6. PubMed https://doi.org/10.1093/bib/bbx108

4. Larsson A. AliView: a fast and lightweight alignment viewer and editor for large datasets. Bioinformatics. 2014;30:3276–8. PubMed https://doi.org/10.1093/bioinformatics/btu531

5. Miller MA, Pfeiffer W, Schwartz T. Creating the CIPRES Science Gateway for inference of large phylogenetic trees. In: 2010 Gateway Computing Environments Workshop (GCE). New Orleans: IEEE; 2010. p. 1–8.

6. Huelsenbeck JP, Ronquist F. MRBAYES: Bayesian inference of phylogenetic trees. Bioinformatics. 2001;17:754–5. PubMed https://doi.org/10.1093/bioinformatics/17.8.754

7. Rambaut A. FigTree. Edinburgh, UK: Institute of Evolutionary Biology, University of Edinburgh; 2018.

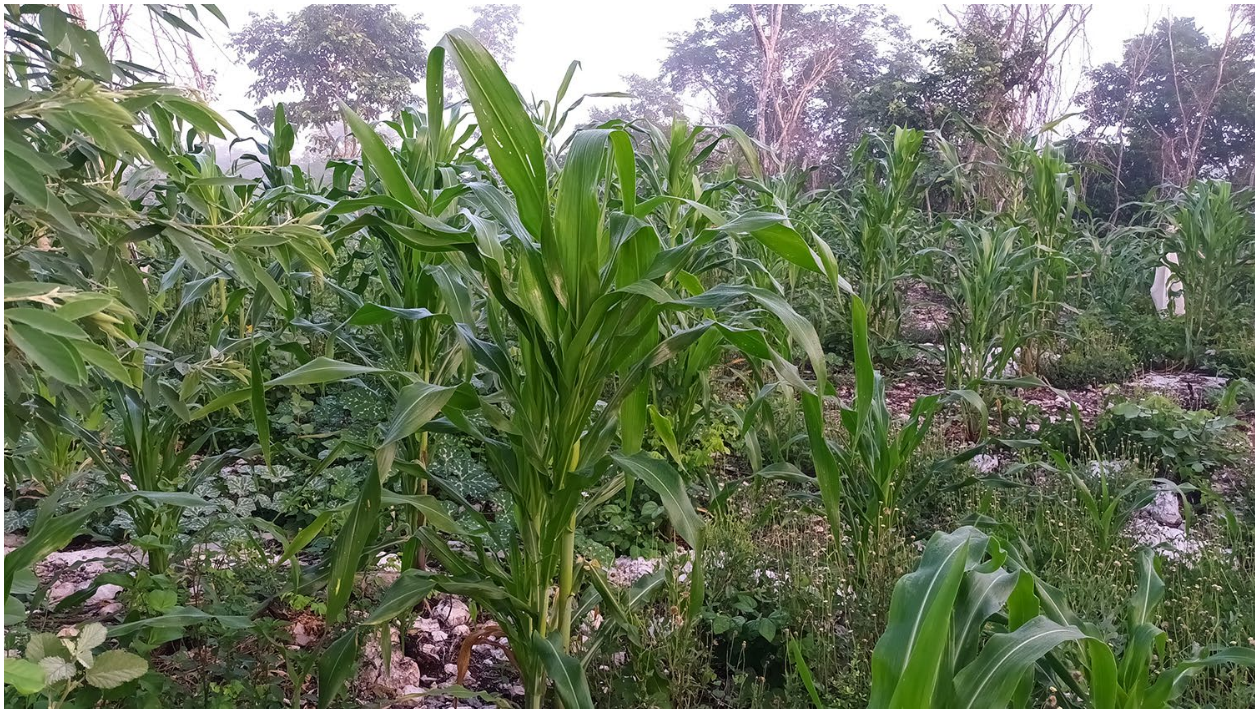

Appendix Figure 1. Ecologic context of field observations of *Aedes* (*Fredwardsius*) *vittatus* in southeastern Mexico. Traditional Mayan cornfield (milpa) on the outskirts of Mama, Yucatán.

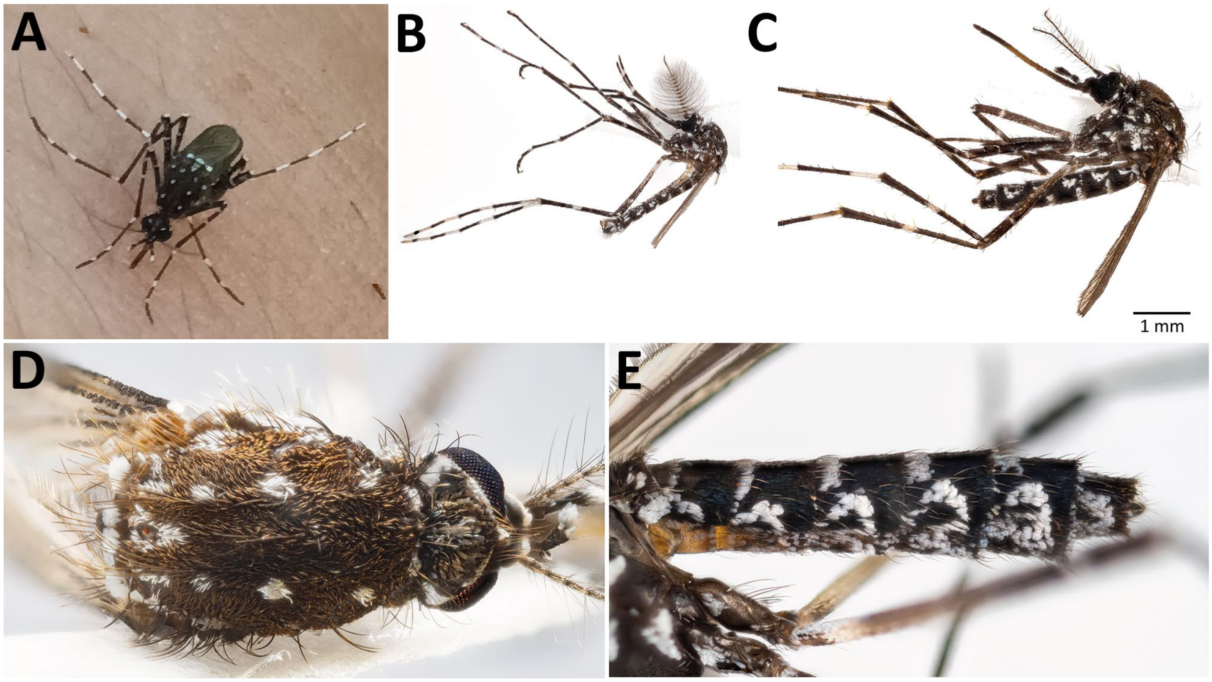

Appendix Figure 2. Morphologic characteristics of *Aedes* (*Fredwardsius*) *vittatus* mosquitoes detected on Yucatan Peninsula, Mexico, 2025. A) Female *Ae. vittatus* mosquito landing on field staff in Mama, Yucatán; B–E) morphologic characteristics of *Ae. vittatus* mosquito specimens: B) male lateral view; C) female lateral view; D) scutum showing narrow white scale patches; E) lateral view of abdomen.

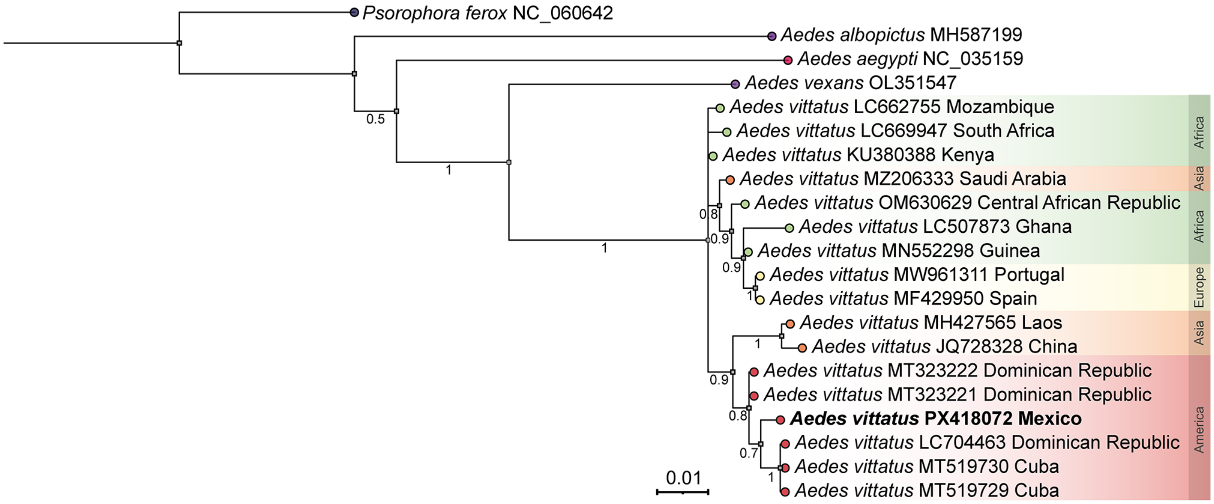

Appendix Figure 3. Bayesian phylogenetic tree comparison of global *Aedes* (*Fredwardsius*) *vittatus* mosquito sequences and those from Yucatan Peninsula, Mexico, 2025. The tree shows the specimen from Mexico (bold) falls within the American–Caribbean clade and clusters with sequences from the Dominican Republic and Cuba. Posterior probabilities are shown at nodes. Scale bar indicates nucleotide substitutions per site.
